# Health of Louisville

**Published:** 1852-08

**Authors:** 


### THE WESTERN JOURNAL.

THIRD SERIES.—VOL. X.—NO. II.

LOUISVILLE, AUGUST 1, 1852.

### HEALTH OF LOUISVILLE.

The diseases of earlier summer have abated. We hear now of but few cases of bowel complaints. Bilious remittent fever is becoming somewha prevalent in the suburbs of the city, and with it cases of intermittent fever are occurring, but in the interior nothing is heard of these diseases. Cholera is not spoken of in any part of the city.

The summer is again one of the hottest; it is indeed seldom that we have two such summers in succession. For a number of days towards the latter end of July, the thermometer ranged from 93° to 96° in a fair exposure.

